# Structure of the Region-Technology Network as a Driver for Technological Innovation

**DOI:** 10.3389/fdata.2021.689310

**Published:** 2021-07-14

**Authors:** Dion R. J. O’Neale, Shaun C. Hendy, Demival Vasques Filho

**Affiliations:** ^1^ Department of Physics, University of Auckland, Auckland, New Zealand; ^2^ Te Pūnaha Matatini—The Centre for Complex Systems and Networks, Auckland, New Zealand; ^3^ Leibniz Institute of European History, Mainz, Germany

**Keywords:** innovation networks, patents, knowledge spillover, agglomeration advantage, bipartite netwoks, patent space, evolutionary economic geography (EEG), technological innovation

## Abstract

Agglomeration and spillovers are key phenomena of technological innovation, driving regional economic growth. Here, we investigate these phenomena through technological outputs of over 4,000 regions spanning 42 countries, by analyzing more than 30 years of patent data (approximately 2.7 million patents) from the European Patent Office. We construct a bipartite network—based on revealed comparative advantage—linking geographic regions with areas of technology and compare its properties to those of artificial networks using a series of randomization strategies, to uncover the patterns of regional diversity and technological ubiquity. Our results show that the technological outputs of regions create nested patterns similar to those of ecological networks. These patterns suggest that regions need to dominate various technologies first (those allegedly less sophisticated), creating a diverse knowledge base, before subsequently developing less ubiquitous (and perhaps more sophisticated) technologies as a consequence of complementary knowledge that facilitates innovation. Finally, we create a map—the Patent Space Network—showing the interactions between technologies according to their regional presence. This network reveals how technology across industries co-appear to form several explicit clusters, which may aid future works on predicting technological innovation due to agglomeration and spillovers.

## 1 Introduction

Innovation is facilitated by the combination of diverse yet complementary knowledge inputs (Jacobs, 1969). Indeed, some of the most influential conceptualisations of the innovation process regard technological change as originating from the combination of new and existing technological capabilities (Weitzman, 1998). A recent study of more than 2 centuries of patents granted in the United States suggests that more than half of all patented inventions in this period arose through novel recombination of pre-existing technologies (Youn et al., 2015).

While the potential for new combinations of the world’s current portfolio of technologies is vast, the economic geography of innovation may constrain the ability of inventors to explore all technological combinations. Furthermore, the value of adding an additional technology to a set of pre-existing capabilities will vary depending on both the new technology and the existing combination. This would suggest that those regions with a diverse knowledge base are at an advantage when it comes to regional technological progress (Feldman and Audretsch, 1999).

The economic geography of innovation is known to be dominated by agglomeration effects and spillovers, where innovation and economic growth are facilitated by geographical proximity (David and Rosenbloom, 1990; Krugman, 1991; Saxenian, 1996) and localized learning processes (Glaeser, 1999). According to Feldman (1999, 5), “knowledge is not easily contained and geography provides one means to define knowledge spillovers,” such that firms, including competitors, can benefit from being locally proximate (Baldwin and Okubo, 2006). In short, agglomeration effects occur when firms or people accrue benefit from being located near to one another, while knowledge spillovers are one process by which firms and individuals can derive such benefits, by taking advantage of new knowledge that has been created by others.

While developments in communication technology make the dissemination of codified knowledge ever faster and cheaper, the transmission of tacit knowledge may still be difficult in the absence of face-to-face interactions. Consistent with this, the effects of knowledge spillovers on innovation have been found to be most evident when people or firms are geographically proximate (Jaffe et al., 1993; Glaeser and Ponzetto, 2010; Baicker and Chandra, 2010; Buzard et al., 2017; Buzard et al., 2020). As a result, inventors who have access to a more highly connected and diverse local innovation system—and the firms that employ them—face lower costs in exploring a larger set of potential technologies.

Knowledge spillovers have been considered to act in two ways. First, concerning firms or inventors within particular industries, spillovers favor *localization*: because of the decay in spillover benefits over distance, similar firms will co-locate so that they can learn from each other (Porter, 1990; Jaffe et al., 1993; Ellison and Glaeser, 1997; Duranton and Overman, 2005; Chatterji et al., 2013). Second, concerning firms or inventors in different industries, spillovers favor *urbanization*: firms from a variety of industries will choose locations where they can benefit from a diverse range of knowledge spillovers (Glaeser et al., 1992).

Regarding the latter, the effect of spillovers weakens across different industrial or technological domains (Jaffe et al., 1993). Due to this, regional innovative performance becomes path dependent: regions are only able to explore new combinations of technologies if the relevant technological and organizational capabilities are already present (Hidalgo and Hausmann, 2009). Thus, our first question is: do regions with a broader knowledge base tend to create high-tech—more unique—innovations than regions with a small set of capabilities? To address that, we have constructed a bipartite *revealed* technological network by observing how specialties appear across geographical regions. Using over 3 decades of data from the European Patent Office we have computed the revealed comparative advantage in specific technological domains for more than 4,000 regions spanning 42 countries. We compare the observed bipartite network with several null models to assess the technology co-occurrence—and possibly knowledge spillover—effects on regional diversity and technological ubiquity. We find that regions with a low diversity of technological capabilities tend to have a more ubiquitous set of technologies than regions with a higher diversity. In other words, technologies that are less ubiquitous (allegedly more sophisticated) tend to occur in regions with high technological diversity. This is consistent with the idea that regional innovation is constrained by access to diverse technological inputs that are available locally.

However, not all co-occurrence and combinations of technologies will have utility [e.g. the “espresso-making toothbrush” (Youn et al., 2015)], while some technologies may lend themselves to many more useful combinations than others (e.g. general purpose technologies, such as the integrated circuits within both smartphones and automobile engines). Then, we pose a second question: how are baskets of technologies organized, thanks to regional output, such that they might favor spillovers and facilitate the prediction of new technologies? From the bipartite network, we have constructed a (projected) network map of technological proximity—based on the co-occurrence of technologies. This approach is similar to that of Hidalgo and Hausmann (2009), who used exported products rather than patents to examine the links between the type of goods exported and economic success at a national level.

The advantage of using patents rather than exported products is that patents can be tied to a particular region, whereas export data is typically aggregated at a national scale. The disadvantage of using patents lies in the greater difficulty in assessing their value: many, if not most patents will have little market value, while a few may be of considerable worth (Hall et al., 2005). Moreover, our approach is complementary to that of Youn et al. (2015), who categorize individual patents by the combination of technological classification codes assigned to them during the application process. However, by using regional co-location of patents as a measure of technological proximity, our method has the potential to capture the incorporation of tacit knowledge in new technologies that would not necessarily be evident in a codified classification scheme. In what follows, we demonstrate that our measure of proximity does differ from a codified classification scheme.

We begin with a brief discussion about the scholarship that underpins this study. Next, we explain the data set used and the methodological approach taken to create the region-technology (bipartite) network, including a description of a variety of null models used to test hypotheses about the spillover effect, and the Patent Space (projected) network of technological co-occurrence in regions. Then, we discuss our results focused on the relationship between diversity and ubiquity found in regional patent portfolios. We conclude with comments on the importance of this relationship.

## 2 Diversification, Path-Dependence and Proximity

Diversity has a long history in theoretical and empirical debates about its effects on several fronts of regional economic development as, for instance, growth, resilience against cycles and unemployment, stability, per capita income, and firms performance (Attaran, 1986; Sherwood-Call, 1990; Malizia and Ke, 1993; Wagner and Deller, 1998; Qian et al., 2008). Under the lenses of evolutionary economics, diversity plays a leading role also in technological progress: due to cumulative and path-dependent processes, regions with a diverse knowledge base are more likely to produce new technologies (Feldman, 1999).

How regions evolve following particular paths is a key question in the recent body of studies dubbed Evolutionary Economic Geography (Boschma and Martin, 2010). On the one hand, the latter draws from evolutionary thinking where the “current state of affairs cannot be derived from current conditions only” (Boschma and Frenken, 2017, 280), characteristic of a path-dependent process: regional capabilities determine which industries—or technologies—are most likely to develop in the future (Hidalgo et al., 2007; Hausmann and Klinger, 2007). On the other hand, evolutionary economic geography builds upon New Economic Geography models concerning agglomeration mechanisms and the formation of dense clusters of industries (Krugman, 1991).

These clusters happen thanks to the advantages of reducing transportation costs, generating economies of scale and easing factor mobility that result in manufacturing belts with a high concentration of people (Krugman, 1991). Ultimately, agglomeration leads to concentrate diversity—through knowledge spillover mechanisms of localization and urbanization (Porter, 1990; Glaeser et al., 1992; Jaffe et al., 1993; Chatterji et al., 2013)—which, in turn, leads to more agglomeration, in an iterative process. Cumulative knowledge (i.e technological output) follows a path of evolution, such that regions that acquire a vast technological base are more likely to produce ubiquitous technologies.

Regional technological innovation, therefore, depends on diversity and spillovers effects. Even though technology has facilitated learning exchange at distance, spillover effects on innovation are most evident with spatial proximity, especially thanks to tacit knowledge (Howells, 2002). However, Boschma (2005) argues that learning and innovation are facilitated by, besides spatial proximity, other four dimensions of proximity—cognitive, organisational, social and institutional proximity. In fact, he says that “geographical proximity per se is neither a necessary nor a sufficient condition for learning to take place: at most, it facilitates interactive learning, most likely by strengthening the other dimensions of proximity” Boschma (2005, 62). Among these other four dimensions, cognitive proximity is more relevant to this study, as it relates to the co-occurrence of technological codes in regions.

Cognitive proximity accounts for how different the knowledge base and the capabilities between two actors is. “The ability to evaluate and utilize outside knowledge is largely a function of the level of prior related knowledge” (Cohen and Levinthal, 1990, 128). Thus, again, we have a path-dependent process of innovation *via* combinations of technologies, the so-called recombinant innovation (Weitzman, 1998; Neffke et al., 2011; Castaldi et al., 2015). Although recombinant innovation is more common within sectors (related to the localization mechanism), it also happens in extra-sector (urbanization) contexts. In the latter case, innovations are more likely to fail but also to be disruptive (Frenken et al., 2007; Castaldi et al., 2015). The Patent Space network we propose attempts to provide insights on these combinations based on regional technological co-occurrence.

## 3 Data and Methods

### 3.1 Data

We use patent records from the February 2016 edition of the Organization for Economic Co-operation and Development (OECD) REGPAT database (PATSTAT), which is itself derived from two complementary sources: the European Patent Office (EPO) Worldwide Statistical Patent Database (PATSTAT, 2018) and the EPO Bibliographic Database and Abstracts (November 2015). The data covers 2,892,607 patent applications filed to the EPO from 1977 to 2012, with partial data from 2012 to 2015.

Patent records in REGPAT are “regionalized” by matching applicant addresses with one of 4,106 micro-statistical regions (TL3) covering the 46 countries in the data set (Maraut et al., 2008). During the filing process, patents are assigned one or more International Patent Classification (IPC) code which attempts to categorize the type of technology that relates to the essential novel component of the invention described in the patent.

The classification system is hierarchical: technologies described by lower levels of the code are subdivisions of the technologies at higher levels. A complete IPC code consists of four levels with additional classification at an additional fifth sub-level in some cases. This divides the technologies described by the patents into roughly 70,000 subdivisions. In the analysis presented in this paper, we use the third level of the IPC codes, consisting of 635 categories.

We provide results with aggregated micro-regions (TL2—639 regions) and the fourth level (IPC4—7,823 codes) of the IPC codes in the Supplementary Material.

### 3.2 Methods

We constructed a matrix of regions and IPC codes where each entry in the matrix is the number of times that a particular IPC code was used on patents from that region. To determine when the count for a particular region-code pair is significant, we used the method of revealed comparative advantage (RCA) (Balassa, 1965):

$$RCA\left(r,c\right)=\frac{x\left(r,c\right)}{\sum_{c}\;x\left(r,c\right)}/\frac{\sum_{r}\;x\left(r,c\right)}{\sum_{r,c}\;x\left(r,c\right)}$$ (1)

where x(r,c) is the number of times code *c* appears on patents filed by an applicant from region *r*.

The RCA method takes account of both the total amount of patenting activity within a region, and the global prevalence of each IPC code as a fraction of all those used in the data. For a given region-technology pair, a RCA value greater than one indicates that the region produces more patents using that technology than would be expected, given the total number of patents produced by that region and the fraction of the world’s patents that also use that technology.

A bipartite network is defined by connecting regions and technology codes when RCA(r,c)≥1. The network can be represented by an unweighted adjacency matrix

$$M_{r,c}=\{\begin{array}{cc} 1, & \text{if}\;RCA\left(r,c\right)\geq 1\\ 0, & \text{otherwise}. \end{array}$$ (2)

**FIGURE 1:**
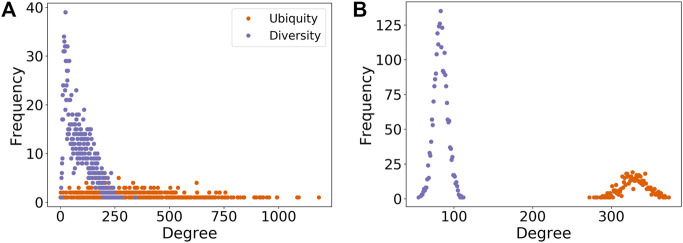
Degree distributions of regions (diversity) and technologies (ubiquity) for the **(A)** empirical and **(B)** random (null model 1) networks. The latter is a bipartite variant of the Erdős and Rényi model (Erdős and Rényi, 1959; Vasques Filho and O’Neale, 2020a). The randomization of connections between regions and IPC codes makes the diversity distribution less right-skewed and changes completely the shape of the ubiquity distribution from a uniform-like to a normal-like distribution. The randomization tests the effects of localization and urbanization (for regions), and sophistication (for technologies), by assuming that there are no underlying attributes of regions and technologies that might be responsible for the observed degree distributions.

The diversity of a region (the number of technology codes for which it has a revealed comparative advantage) is given by $d_{r}=\sum_{c}M_{r,c}$, while the ubiquity of a technology code (the number of regions which have a revealed comparative advantage with respect to that code) is given by $u_{c}=\sum_{r}M_{r,c}$. These correspond to the degrees of the region and technology nodes, respectively, in the bipartite network (Figure 1A). For each region, we also calculate the average ubiquity of the technologies for which that region has RCA≥1: ${\langle u\rangle }_{r}=\frac{1}{d_{r}}\sum_{c}M_{r,c}u_{c}$.

#### 3.2.1 Bipartite Network Null Models

In order to determine whether the structure we observe in the region-technology network diverges from that where regional and/or technological attributes are ignored, we compared its properties with those from random networks produced by five different null models. We associate each null model with a particular hypothesis about what effects may be causing the observed structure. Four null models rewire the empirical region-technology network in different ways, based on Hidalgo and Hausmann (2009). The, fifth null model generates alternative region-technology networks by randomly reallocating to regions the patents, as recorded in the PATSTAT database. this accounts for the co-occurrence of multiple technology codes on the same patent. For each of the null models we run 100 realisations. Below we describe these five models and the hypothesis we test with them.

• **Model 1:** Randomly reassigns the edges in the network conserving only the number of nodes and number of edges. This is a bipartite variant of the Erdős and Rényi model (Erdős and Rényi, 1959; Vasques Filho and O’Neale, 2020a). The model preserves the mean degree for the two node types, but not the degree of individual nodes neither the shape of the degree distribution (Figure 1B). It has the highest level of randomization, meaning that regions and technologies have similar levels of diversity and ubiquity, respectively. Here, we test the effects of localization and urbanization in regions and sophistication of technologies due to recombination at the same time, by ignoring the attributes of both region (e.g. population, number of research institutions) and IPC codes (e.g. different amounts of capability or resources required for their development) related to these effects.
• **Model 2:** Randomly reassigns edges while preserving the degree sequence of regions (i.e. preserving $d_{r}$ for each region node and the diversity distribution of Figure 1A), in addition to preserving the total numbers of nodes and edges. This model can be interpreted as treating all technologies as being identical, while each region possesses some property (and the localization and urbanization effects) that affects its ability to develop a RCA in any technology.
• **Model 3:** Randomly reassign edges while preserving the degree sequence of technology codes (i.e. preserving $u_{c}$ for each code and the ubiquity distribution of Figure 1A). Such a model represents the situation where all regions are identical, but technologies each posses some attribute that affects the likelihood of them being employed in any invention. This would be consistent with the hypothesis that some technologies are easier to acquire and hence are more prolific than others.
• **Model 4:** Randomly swap pairs of existing edges such that for a pair of edges (a,b) and (u,v) they are replaced by (a,v) and (u,b) if neither of the two new edges were already part of the network. This is the most stringent of the rewiring models, preserving the degree sequences of both the region and technology nodes (as in Figure 1A), but removing correlations that might exist between specific technologies and their associations with specific regions.
• **Model 5:** Randomly reassign individual patents to regions, such that each region retains the same total number of patents, before calculating a new bipartite network. Individual patents can, and often do, list more than one technology code. This model preserves the co-occurrence of these codes on patents, but not co-occurrence of technology codes due to regional clustering.

#### 3.2.2 The Patent Space (Projected) Network

The five null models each test the relationship between the diversity of technologies used by a region for its inventions and the ubiquity of those technologies. However, while they do test the observed structure of the region-technology network as a result of agglomeration or spillover effects, they do not explain how these effects occur.

To investigate that, we look at the co-occurrence of different technologies within regions by projecting the region-technology network to get a technology-technology co-occurrence network. For this projection, we follow the example of Hidalgo et al. (2007) who constructed a similar network for products exported by different countries. The proximity (weight of the edge) between any pair of IPC technology codes is given by the pairwise conditional probability that a region with RCA≥1 for code $c_{i}$ also has RCA≥1 for code $c_{j}$. that is

$$\phi \left(c_{i},c_{j}\right)=\min\left\{P\left(RCA\left(c_{i}\right)\geq 1|RCA\left(c_{j}\right)\geq 1\right),P\left(RCA\left(c_{j}\right)\geq 1|RCA\left(c_{i}\right)\geq 1\right)\right\}$$ (3)

## 4 Results and Discussion

### 4.1 Empirical Data

**FIGURE 2:**
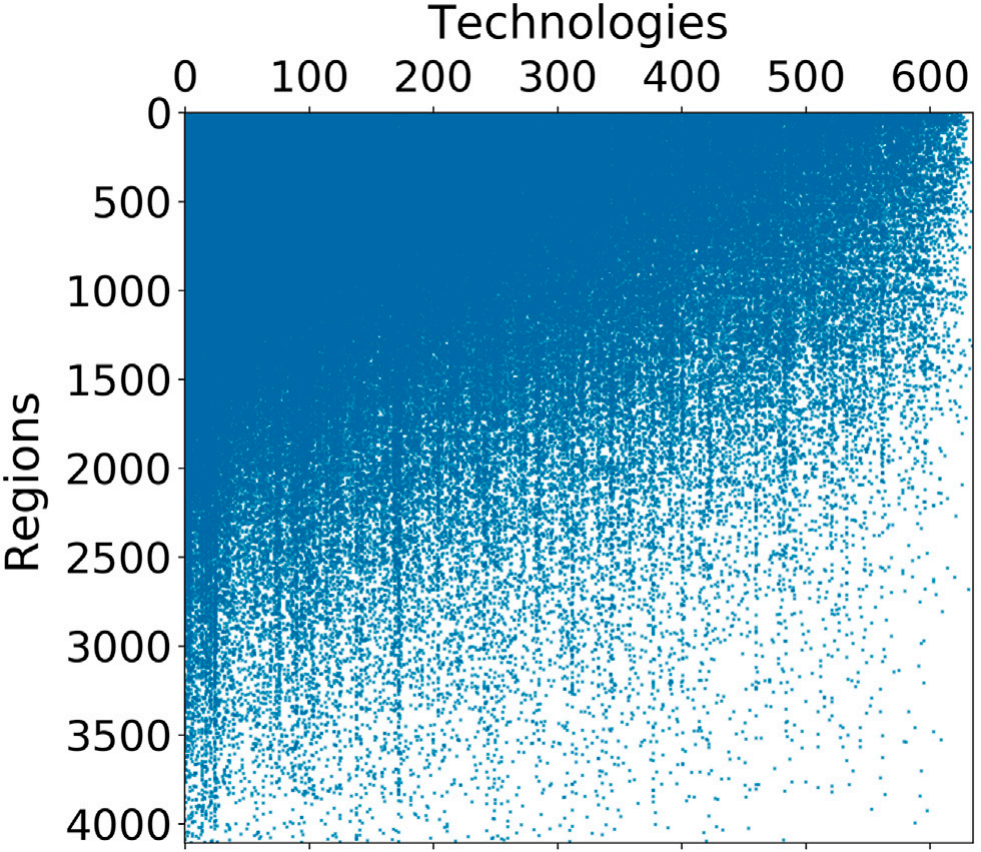
Adjacency matrix of the region-technology network ordered by degree value, from highest to lowest diversity **(top–bottom)** and ubiquity **(left–right)**. The triangular-like structure tells that high diversity regions file patents with both low and high ubiquity IPC codes, while low diversity regions are only able to make use of the most ubiquitous—and presumably less sophisticated—technologies.

We can begin to understand the distribution of technical capabilities among regions by looking at the structure of the adjacency matrix $M_{r,c}$. Ordering the rows and columns of the matrix according to the values of $d_{r}$ and $u_{c}$ shows that the matrix is approximately triangular (Figure 2).

The triangular structure implies that more technically advanced regions—those with high diversity—file patents involving both low and high ubiquity technologies, while low diversity regions are only able to make use of the most ubiquitous—and presumably less sophisticated—technologies. Thus, the triangular structure indicates that the technologies associated with inventions from low diversity regions tend to be subsets of those used by high diversity regions. Also, this structure indicates that the Ricardian model of producing goods for trade does not apply here, if we were to extrapolate the model for the production of technology. Such a model for technological production would lead to an adjacency matrix with a block-diagonal structure as regions would specialize in only those technologies that they have a comparative advantage for and which will have the lowest ubiquity, at the expense of having high diversity through also producing some more ubiquitous technologies.

A consequence of the triangular structure for the adjacency matrix is that those regions with higher diversity $d_{r}$ tend to have a lower mean ubiquity ${\langle u\rangle }_{r}$. In the case of a perfectly triangular m×n adjacency matrix

$$M_{r,c}=\{\begin{array}{cc} 1, & \text{if}\;r/c\leq m/n\\ 0, & \text{otherwise}, \end{array}$$

it can be shown analytically that mean ubiquity ${\langle u\rangle }_{r}$ is a linear function of diversity $d_{r}$, given by

$${\langle u\rangle }_{r}≃-\frac{m}{2n}d_{r}+m.$$

**FIGURE 3:**
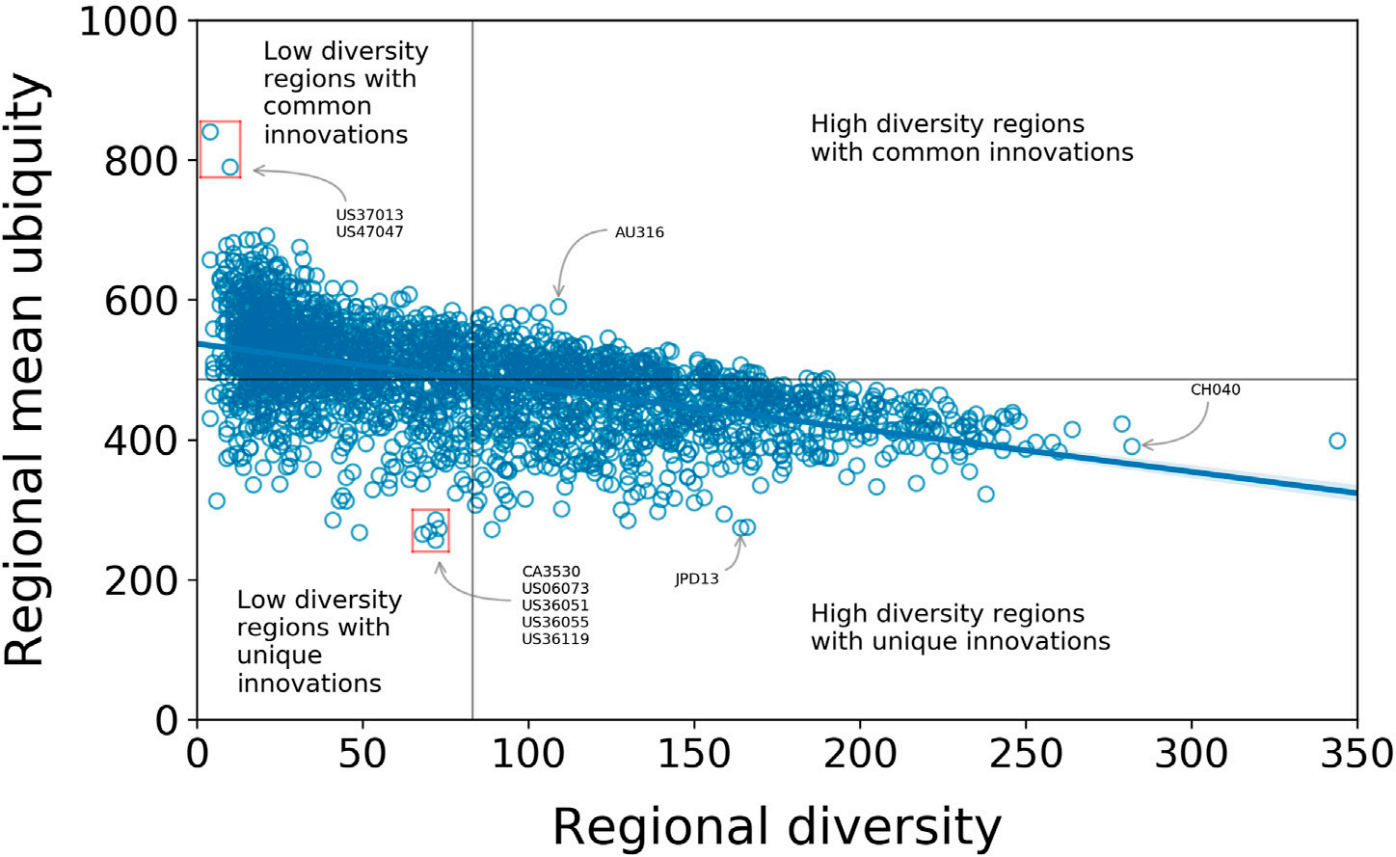
Correlation between regional mean ubiquity and diversity of regions with Pearson correlation coefficient r=−0.52 ($R^{2}=0.27$). We partition the regions into those with high diversity and relatively unique technologies, and those with low diversity and common technologies and give some examples of regions in these quadrants.

The empirical correlation between diversity $d_{r}$ and regional mean ubiquity ${\langle u\rangle }_{r}$, with a linear least squares fit, gives a slope of −0.61 and an intercept of 537.0. The Pearson correlation coefficient is r=−0.52 ($R^{2}=0.27$) (Figure 3). While the $R^{2}$ value is relatively low, this is to be expected since, particularly for regions with only a small number of patents, there will be a range of factors that influence the likelihood of a technology being recorded in a region. (To avoid more of this noise, we make this analysis considering only the regions with more than ten patents in the whole period. This is a rather weak constraint that filters out only those regions which have close to zero patenting activity over the 30 year period of the study data.) The nature of this correlation makes it possible to approximately partition the regions into those with high diversity and relatively unique technologies, and those with low diversity and common technologies (Figure 3).

**FIGURE 4:**
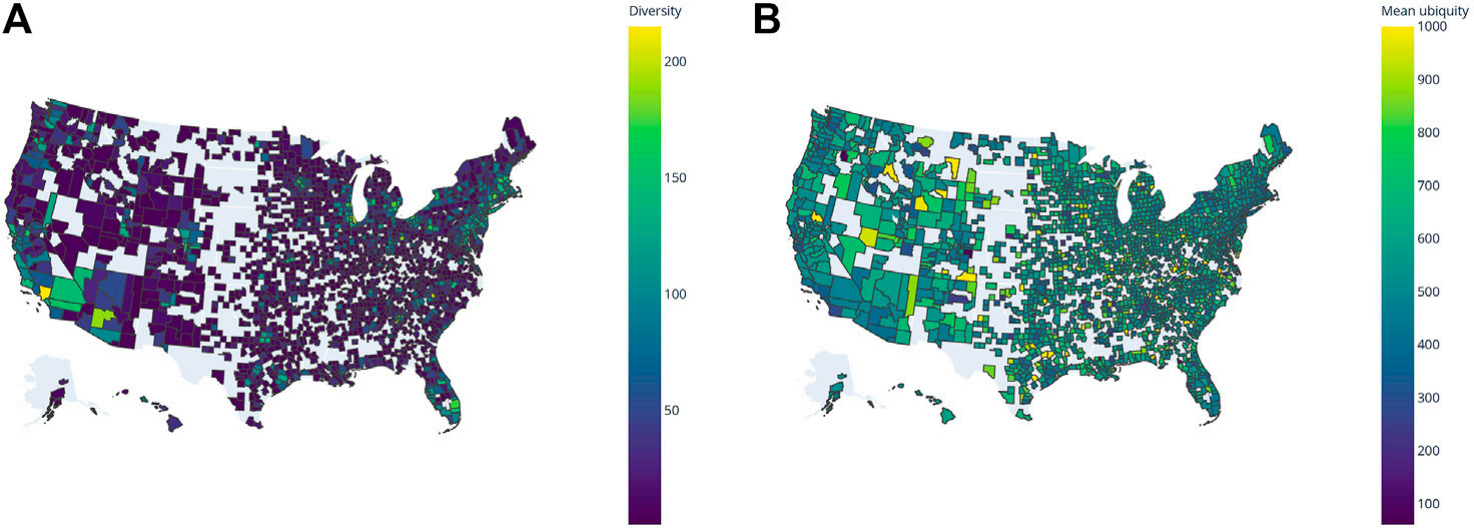
Spatial autocorrelation of counties in the United States. **(A)** High-diversity regions tend to be closer together, as seen in California, Florida and the Northeastern corridor. **(B)** Mean ubiquity is higher for those less diverse regions, which are usually more distant from high-diversity ones.

While we do not carry out a detailed analysis of the spatial distributions of technologies and the correlation between them, we would like to highlight some high-level trends using some examples, especially in the United States. From the about 3,200 United States counties, almost 2,100 have a RCA in at least one IPC code, and 90% of those are in either one of the two low-diversity quadrants of Figure 3. Some (as Beaufort County, South Carolina and Fayette County, Tennessee) have high mean ubiquity, implying these regions produce a small set of low-tech innovations; others have low mean ubiquity (as San Diego, in California, and Livington, Monroe and Westchester counties in New York), suggesting they present more sophisticated innovations as part of their technological output. The former are usually far from high diversity regions in contrast to the latter, which are closer (Figure 4). This pattern also indicates a clustering of regions with similar profiles. That is, high-diversity regions tend to be geographically proximate to one another, and next to them regions with both low diversity and mean ubiquity tend to appear. This idea is supported in a global scale as the tendency of high-diversity regions having a RCA in less ubiquitous technology holds when regions at the TL3 level are aggregated at the TL2 level (Supplementary Material).

On the quadrants with high diversity in Figure 3, there is a small fraction of regions with high mean ubiquity (e.g., Sunshine Coast, in Australia), and several with low mean ubiquity (e.g., Tokyo and Zurich).

### 4.2 Null Models

**FIGURE 5:**
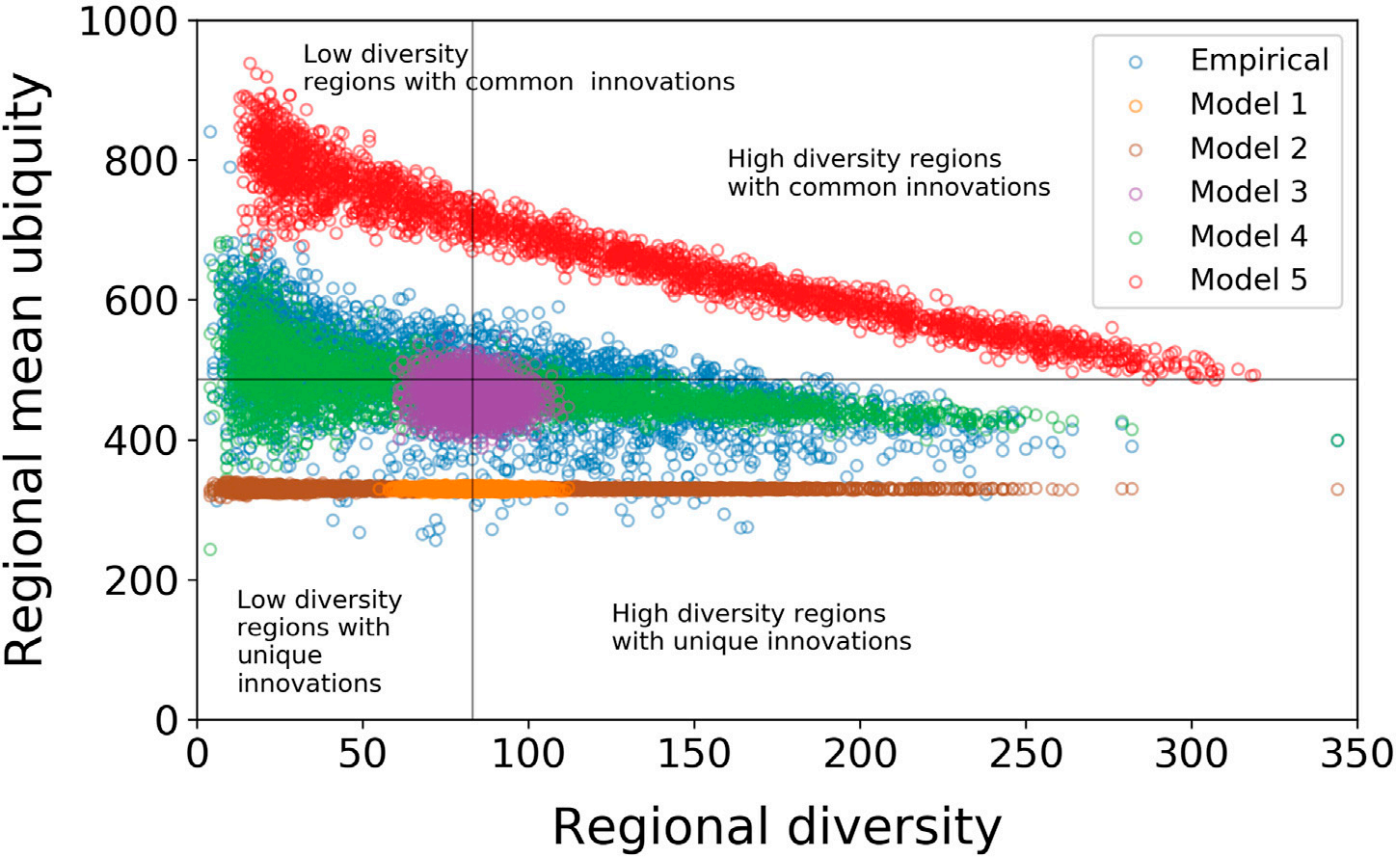
Null models 1, 2, and 3 (random rewiring and preserving the degree sequences of the technology or the region nodes) completely destroy the negative relationship between diversity and mean ubiquity that is observed in the empirical data. Null models 4 (preserving both degree sequences) and 5 (reallocating patents) manage to qualitatively reproduce the relationship in the empirical data, but are a poor quantitative fit, suggesting that there are addition effects such as co-occurrence of particular combinations of technologies, alongside agglomeration and spillover effects resulting in regional specialization that are driving part of the observed pattern.

As expected, the correlations of the null models behave differently from that of the empirical data, with the differences increasing with the level of randomization (Figure 5). In null model 1 (orange rings in Figure 5), where regions and technologies are connected completely at random, just keeping the total number of connections, all regions have similar diversity (as shown in Figure 1B) and virtually the same regional mean ubiquity. The latter is significantly lower than the average regional mean ubiquity in the empirical data thanks to all technologies having similar ubiquity after rewiring (Figure 1B). The combination of low-diversity regions with high-ubiquity IPC codes that brings mean ubiquity up do not exist when all codes have similar ubiquity. These observations are indications that knowledge spillover might be affecting regional and technological attributes through localization and urbanization of regions, and recombination of technological codes, respectively.

With null models 2 and 3, we decrease the level of randomization and test separately IPC codes co-occurrence effects on technological and regional characteristics, respectively. On one hand, null model 2 (brown rings in Figure 5) preserves only the diversity of regions. Again, we see that regions have similar regional mean ubiquity thanks to all technologies being treated as equals, in terms of their acquisition by regions. That is, regional attributes alone (represented by regional diversification) cannot explain technological ubiquity and regional mean ubiquity as seen in the empirical data. On the other hand, null model 3 (purple rings in Figure 5) preserves the ubiquity of technologies and ignores regional attributes. The model results in what resembles a two-dimensional normal distribution for regional diversity and mean ubiquity. Thus, technological ubiquity cannot explain these two variables by itself.

Then, in null model 4, we preserve both regional diversity and technological ubiquity but rewire the links between regions and technologies. In this case, the correlation between regional diversity and mean ubiquity is much closer to the empirical data than the previous null model (green rings in Figure 5). It becomes meaningful again look at the statistics of the correlation. In this case, the linear least squares fit gives a mean slope (95% CI) of −0.35(−0.33<m<−0.37) and an intercept of 505.2(502.5<c<507.8) over 100 realisations. The Pearson correlation coefficient is r=−0.51 ($R^{2}=0.26$). The intercept and the correlation coefficient are very similar to those of the empirical data, however the slope is significantly lower than the −0.61 for the empirical data. We credit this difference in the slope to the possible correlations between specific technologies and their associations with specific regions not captured when rewiring the links. That is, even when we preserve regional diversity and technological ubiquity in the network, the model still does not capture the path-dependence of specific regions specializing in certain technologies and the clustering of technologies that can result from localized knowledge transfer and agglomeration.

Finally, each patent can be associated with more than one IPC technology code. It is therefore necessary to also test whether the structure we observe can be explained by the co-occurrence of technologies on patents, rather than co-occurrences within regions. Null model 5 (red rings in Figure 5) differs from the four rewiring null models in that it randomly reassigns the existing stock of patents to regions. It is the most stringent of the null models we apply; it preserves the co-occurrence of IPC codes on patents and allocates each region the same number of patents that it originally held. As with null model 4, the results of this model are qualitatively similar to the empirical data. The linear least squares fit gives a mean slope (95% CI) of −1.12 (−1.10<m<1.14) and an intercept of 816.4 (811.7<c<821.0). The Pearson correlation coefficient is r=−0.96 ($R^{2}=0.92$). We attribute these and the increase in the regional mean ubiquity to the model not preserving any regional specialization in technologies. Hence, all technologies are more ubiquitous than they would be if certain regions were more likely to be allocated patents with particular IPC codes, according to regional specialization.

### 4.3 Co-occurrence of International Patent Classification Codes

In order to better explain the features of the regional structure of technical innovation that are unaccounted for by null models four and five, we now look at the co-occurrence of technologies within regions and the associated network of technological dependency that this implies.

The co-occurrences of technologies that result from regional specialization were used to define a network of the IPC technology codes, with connections between pairs of codes when they tended to co-occur within regions more than would be expected by chance. This network of technologies can be thought of as a projection of the region-technology network, where the weight of the edges in the projection is defined by Eq. 3. In analogy with the network in (Hidalgo et al., 2007), we refer to the resulting network of technology codes as the Patent Space.

**FIGURE 6:**
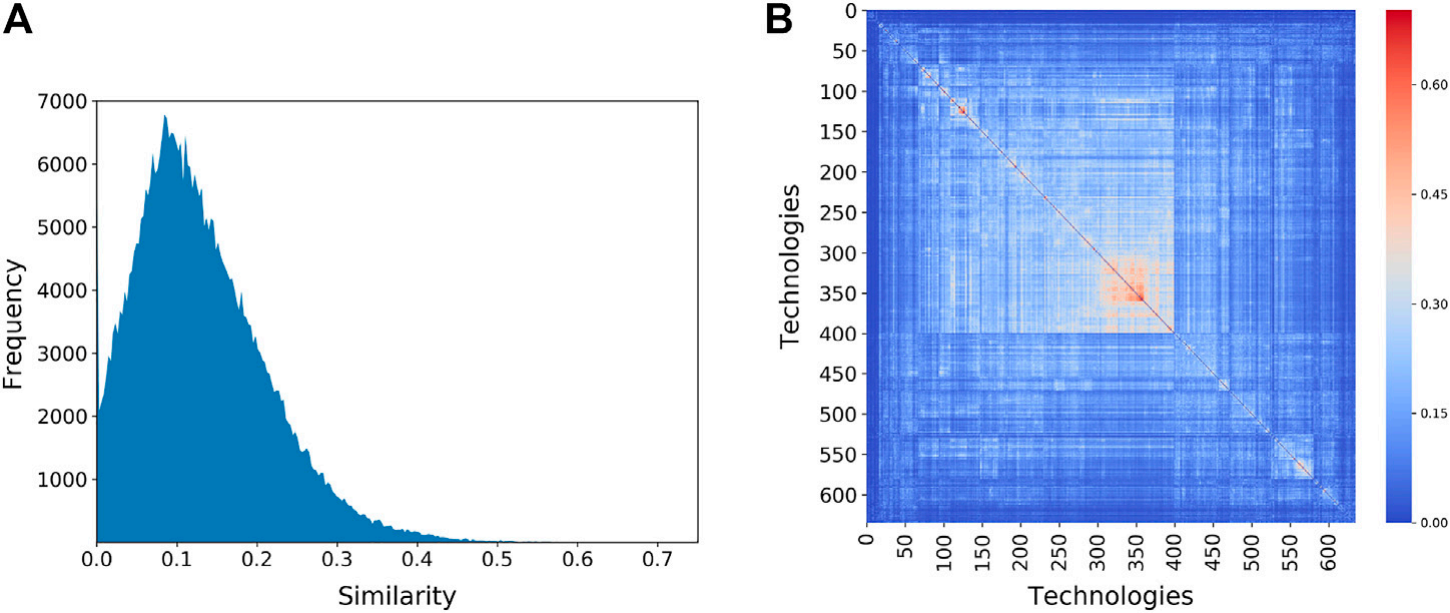
**(A)** Distribution of proximity values between technologies showing most of the edges in the network have low weight. **(B)** Clustering of technologies within regions, as measured by the proximity between technologies. Each row/column represents one of the 632 technology codes. “Hotter” colors indicate a higher value for the proximity between technology codes. The rows and columns of the proximity matrix have been reordered to reveal the clustering of technologies.

The Patent Space network is highly connected—around 98% of all possible proximity links are present. This is an expected structure thanks to the high diversity of several regions that create large clique of codes (Vasques Filho and O’Neale, 2018; Vasques Filho and O’Neale, 2020b). Most links, however, are rather weak due to the projection method: the mean proximity (mean edge weight) between technologies is only 0.125 (the distribution of these values is shown in Figure 6A). The block diagonal structure of the proximity matrix reveals clusters of technologies that tend to co-occur within the same sets of regions. These are indicated in Figure 6B where the rows and columns of the proximity matrix have been reordered so as to maximize the clustering.

**FIGURE 7:**
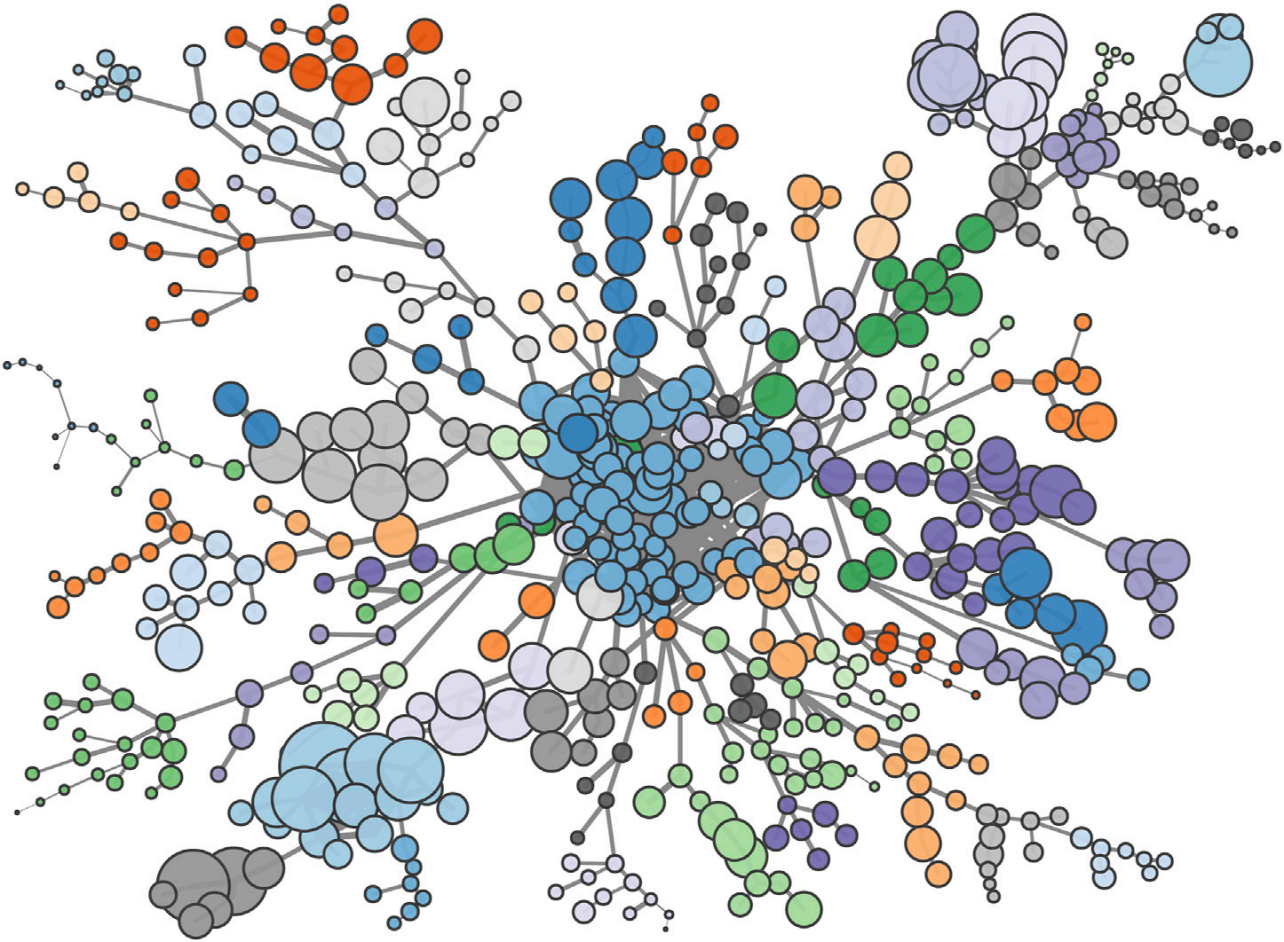
Patent-space: nodes indicate the 632 IPC technology codes, linked by their likelihood of co-occurring within a geographic region. Node sizes are proportional to the number of times each code appears while colors indicate communities as determined by a modularity maximizing community detection method.

The large number of weak connections in the proximity matrix means it is practical to visualize the Patent Space network. We do so by first extracting the maximal spanning tree of the full network and then adding in all those proximity links above a certain threshold. In Figure 7 we use a threshold of ϕ≥0.4, which gives a network with an average degree of around four. This threshold was chosen primarily for visual clarity; smaller or larger values will result in a similar network with more or less detail.

We applied a modularity maximizing community detection method (Rosvall and Bergstrom, 2008) to the network in Figure 7. This partitions the 632 technology codes into around 80 distinct communities. The largest of these contains around 75 (12%) of the technology codes and is located at the center of the network. It presents all sort of technologies, including agriculture, animal husbandry, dentistry, furniture, ceramics, treatment of water, and so on. The remaining communities are roughly an order of magnitude smaller and typically are associated with a particular industry, application or area of technology, even when the IPC codes within that community come from different branches of the IPC hierarchy. Furthermore, the clusters of technologies that relate to a common industry tend to be proximatly located on the network; for example the large branch on the upper left of the network contains a number of technology clusters that are all associated with fiber and textile processing, or derivative products. However, some communities, especially of chemicals, can present a surprising mix due to their wide range of applications as, for instance, a community with technologies related to preservation of bodies (human, animals or plants) and adhesive materials.

Although we cannot infer causation, the heterogeneous network structure implies that there is a non-trivial clustering of specific technologies within geographic regions, consistent with the expected effects of both technological spillovers and agglomeration.

## 5 Conclusion

In this paper we used meta-data on the geographic location of patent applicants, and the technologies specific to the patents they filed, to investigate the regional structure of technological innovation. Using revealed comparative advantage as a metric to identify when regions produced a greater than expected number of patents related to a particular technology, we constructed a network linking regions to technologies.

The adjacency matrix associated with this network can be re-arranged to give a roughly triangular structure. This indicates that regions continue to produce patents relating to ubiquitous technologies even when they are producing patents that use technologies accessed by few other regions; a behavior that is inconsistent with a Ricardian model of technological specialization. We found that those regions which have a high diversity of technologies present in their patent portfolio tended to have a lower mean ubiquity of those technologies, relative to less technologically diverse regions. That is likely related to cumulative and path-dependent processes, characteristic of the evolutionary economic geography framework.

Examples of regions with high diversity are Zurich; Nord-Pas de Calais, a port/industrial area in north France, bordering the English Channel and Belgium; Gauteng, the province where Johannesburg is situated; Bern; Cologne; and Torino. On one hand, ubiquitous technologies include containers for storage (bags, barrels, bottles, etc.); treatment of water, waste water, and sewage; general building construction, such as walls, roofs, insulation and others; shaping of plastic materials; and transporting or storage devices. On the other, some low ubiquity technologies are related to energy production, as fusion and nuclear reactors; computing (e.g neural networks for image processing, cryptography, sensors); and aerospace activity.

The negative correlation between the diversity and mean ubiquity of the technologies in a region’s patent portfolio is not simply a consequence of properties of the individual regions (e.g. population) of technologies (e.g. difficulty to access). Re-wiring null models that take account of such effects are insufficient to explain the observed structure. Nor is the structure simply due to the co-occurrence of multiple technology codes on individual patents. Randomizing which patents are assigned to which regions causes a significant increase in the mean ubiquity of the patent portfolia of all regions.

The null models suggest that the observed structure is due to co-occurrence of different technologies within geographic regions. The structure of the co-occurrences defines a network of technologies that tend to be located within the same sets of regions, related to the cognitive proximity of actors present in these regions. This network has a heterogeneous structure with a core of ubiquitous technologies surrounded by branches with communities of related technologies, associated with specific industries and product types. This non-trivial structure suggests that spillover and agglomeration effects are involved in the distribution of technological innovation. The Patent Space network gives a powerful tool for understanding the role that combinations of knowledge play in determining the success of innovation activities, and hence the economic prosperity, of regions.

## Data Availability

Publicly available datasets were analyzed in this study. These data can be found here: https://data.epo.org.
