# Supplementary material for: Structure of the Region-Technology Network as a Driver for Technological Innovation
Source: Front Big Data. 2021 Jul 14;4:689310. doi: 10.3389/fdata.2021.689310 (PMC8316829; doi:10.3389/fdata.2021.689310)
Supplement: Supplementary file 1 [file DataSheet1.PDF]

Supplementary Material — Structure of the region-technology network as a driver for technological innovation

1 SUPPLEMENTARY FIGURES

Here, we provide the empirical analysis of the main text for two different situations. First, we use the aggregate level (TL2) of the 4,106 micro-regions, which results in 639 regions. We keep the codes at the IPC3 level (635 categories). Second, we use the micro-region level (TL3) with the fourth level (IPC4) of technological code, resulting in 7,823 categories. These variations are done to check how robust the results in the main text are at different scales of regional and technological definitions.

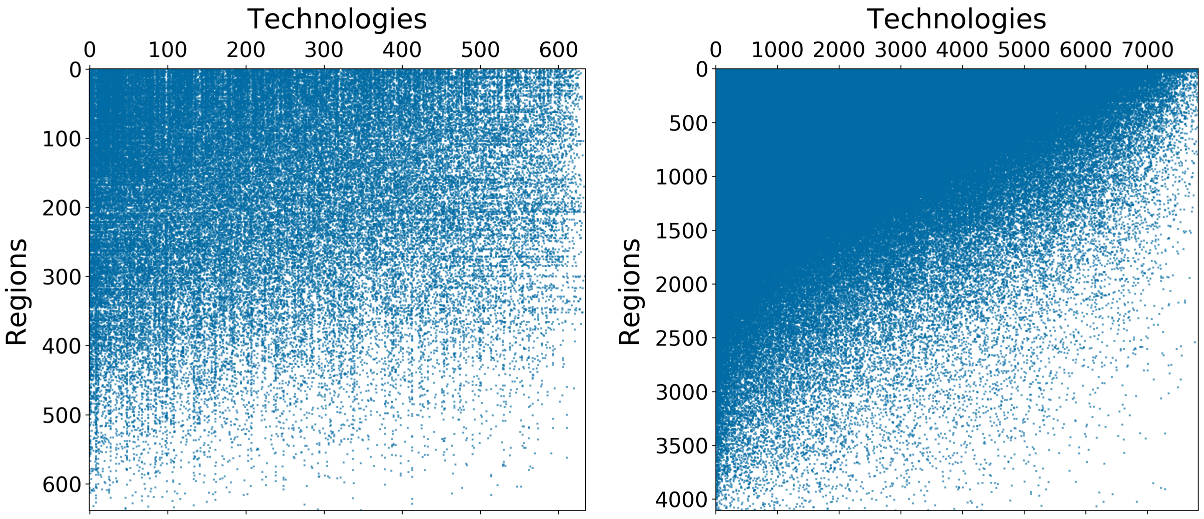

Figure S1. Adjacency matrices of the region-technology network ordered by degree value, from highest to lowest diversity (top–bottom) and ubiquity (left–right). Left: with regions aggregated at the TL3 level, diversity and ubiquity increase overall. However, we still see the resemblance of the triangular shape of the adjacency matrix, with many regions and technologies with low diversity and ubiquity, respectively. Right: IPC codes at a more fine-grained level (IPC4) show a even starker triangular structure.

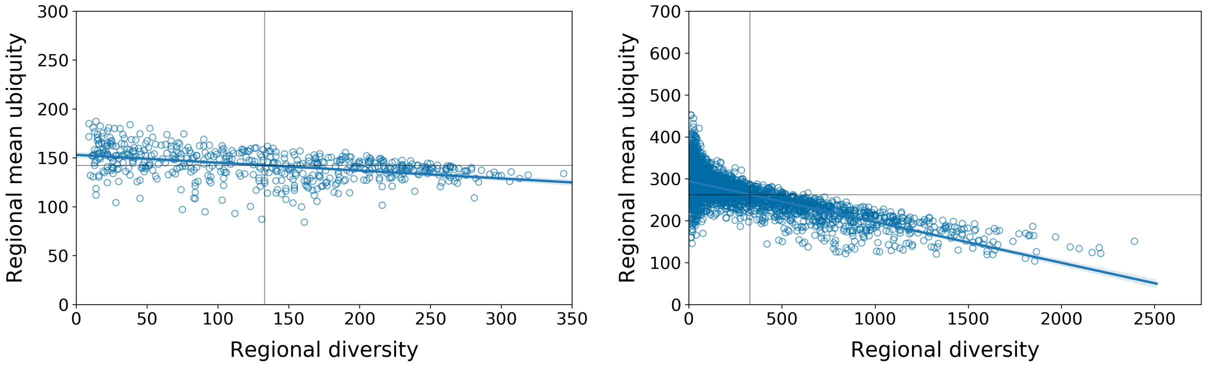

Figure S2. Correlation between regional mean ubiquity and diversity of regions. Left: although the slope of the correlation becomes much less steep with aggregated micro-regions (-0.08), it is still negative, showing the tendency of more diverse regions possessing less ubiquitous technologies. The Pearson correlation coefficient is $r = -0.41$ ($R^2 = 0.18$). Right: for a more granular level of IPC codes, the slope is again much smaller and still negative (-0.09). The higher Pearson correlation coefficient is $r = -0.73$ ($R^2 = 0.53$).

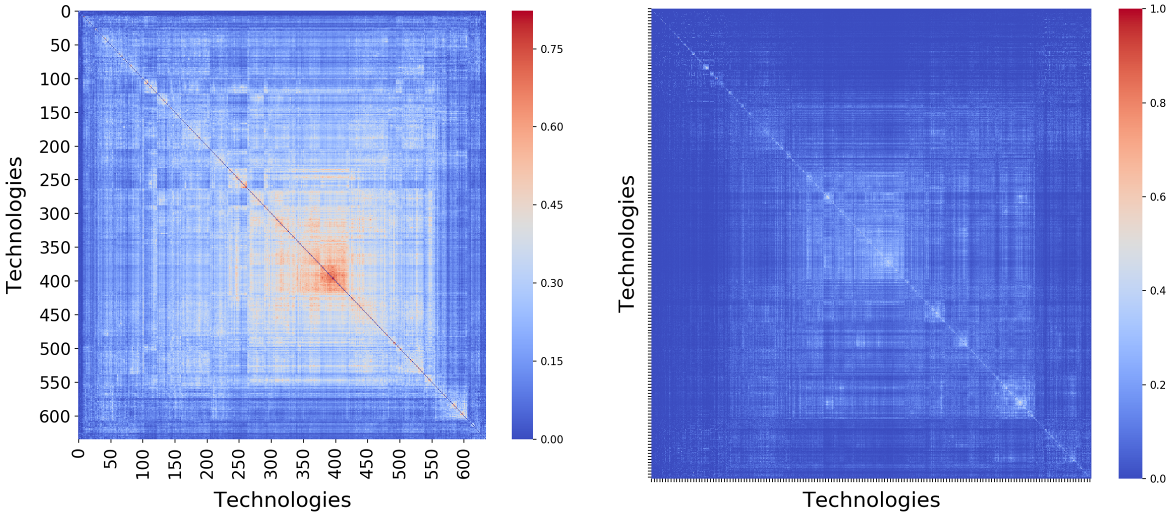

Figure S3. Clustering of technologies within regions, as measured by the proximity between technologies. Left: we can still see strong geographical clustering of technologies even at the scale of regions. Right: as expected, the clustering is much weaker at the IPC4 level, as the technological categories become too granular.
